# Comparative analysis of immune responses to intraperitoneal administration of lytic *E. coli* bacteriophages in mice

**DOI:** 10.1016/j.virusres.2025.199610

**Published:** 2025-07-19

**Authors:** Madina S. Alexyuk, Andrey P. Bogoyavlenskiy, Irina A. Zaitseva, Elmira S. Omirtaeva, Yergali S. Moldakhanov, Kuralay S. Akanova, Elmira I. Anarkulova, Vladimir E. Berezin, Pavel G. Alexyuk

**Affiliations:** Research and Production Center for Microbiology and Virology, Almaty, Kazakhstan

**Keywords:** Bacteriophage, *E. coli*, Immune response, Gene expression, Interleukins

## Abstract

With the global spread of bacterial resistance and the decreasing effectiveness of antibiotics, the relevance of phage therapy is constantly growing. However, for systemic use of bacteriophages in medical practice, a complete understanding of their interaction not only with bacterial cells but also with human and animal organisms in the context of co-administered therapy is required.

This article presents the studies into the effects of lytic *Escherichia coli* bacteriophages on various immune response factors after their intraperitoneal administration in mice. It was found using Real-Time PCR and ELISA methods that the administration of the vB_EcoM_SCS4 and vB_EcoM_SCS57 phages did not increase the gene expression of TLR3, TLR9, IL-4, IL-5, IL-6 and the concentration of IL-2, IL-4, IL-5 and IL-6 in mice, but led to a multi-fold increase in the gene expression and concentration of IFNγ. Whereas, the injection of the vB_EcoS_SCS44 phage into mice increased the expression of the studied genes, except for the IL-5 gene, by 4 to 7 times and increased the concentration of IL-2, IL-4, IL-6 and IFNγ, except for IL-5, by 2 to 3 times. When determining the titer of virus-specific antibodies, it was found that after the administration of phages vB_EcoM_SCS4 and vB_EcoM_SCS57, the titer of IgA, IgG and IgM did not differ from that of the control animals, but the administration of phage vB_EcoS_SCS44 stimulated a twofold increase in the titer of phage-specific IgA, IgG, IgM.

## Highlights

• Lytic bacteriophages are a promising alternative to ineffective antibiotics.
• In order to introduce phage therapy into widespread practice, studies on the effect of phages on patient immunity are needed.
• The lytic phages *Escherichia coli* vB_EcoM_SCS4 and vB_EcoM_SCS57 were unable to significantly affect the immune responses of mice.
• The lytic phage vB_EcoS_SCS44 was able to stimulate factors of innate and adaptive immunity in mice.

## Graphical abstract

**Figure:**
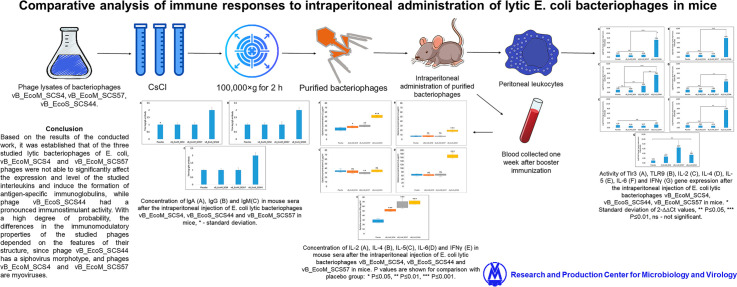

## 1 Introduction

*E. coli* is a widespread commensal Gram-negative bacteria inhabiting the gastrointestinal tract of humans and animals. However, among all the safe *E. coli* strains, there are pathogenic serotypes that can be causative agents of intestinal infections, urinary tract infections, soft tissue and central nervous system infections, and can cause the development of peritonitis and pneumonia (Croxen and Finlay, 2010; Cabal et al., 2016; Nicolas-Chanoine et al., 2014; Montso et al., 2019). Pathogenic *E. coli* strains are particularly dangerous for hospitalized immunocompromised patients (Russo and Johnson, 2003; Manges et al., 2019).

The main way to combat pathogenic forms of *E. coli* is antibiotic therapy, but amid constant contact with humans and their activities, a large number of *E. coli* strains have developed resistance to almost all known classes of antibiotics (Gould et al., 2013; Barilli et al., 2019; Alexyuk et al., 2025). This circumstance significantly reduces the effectiveness of therapy.

Under such conditions, the danger of persistent nosocomial infections is becoming alarming, and studies to find alternative ways to combat them are becoming increasingly important. Bacteriophages are considered to be the most promising alternative to antibiotics (Hitchcock et al., 2023; Jassim and Limoges., 2014).

The advantage of bacteriophages is that they possess high specificity, which allows preserving the beneficial microflora during their application. They are also non-toxic and harmless, have the ability to self-reproduce in the presence of the host bacterium, and can penetrate into bacterial biofilms and lysate them (Górski et al., 2018; Chibeu et al., 2012).

After a significant decrease in the effectiveness of antibiotic therapy over the past couple of decades, interest in the study and application of phage therapy has increased considerably. There are numerous examples of successful use of bacteriophages as therapeutic agents for the treatment of antibiotic-resistant bacterial infections (Gordillo Altamirano and Barr, 2019; Chang et al., 2018; Kortright et al., 2019; Alexyuk et al., 2023).

However, as in all such cases, the use of bacteriophages in human therapy requires a careful and cautious approach. In particular, it is necessary to know how the immune system of patients receiving bacteriophages will react to them.

In addition, approximately 10^16^ phage particles are already present in each person's body. Most of these phages are found in the natural habitats of bacteria: the digestive tract, genitourinary system, respiratory tract and skin (Nguyen et al., 2017). Naturally, bacteriophages influence the formation of the human microbiome, its species diversity and act as a factor in the storage and transfer of bacterial genetic material, which leads to indirect, both negative and positive, effects of phages on human immunity (Ogilvie and Jones, 2015). Many lysogenic phages are known to encode proteins responsible for bacterial virulence factors that allow bacterial cells to penetrate tissue barriers and promote their adhesion, colonization and biofilm formation (Waldor and Mekalanos, 1996; Secor et al., 2015). In these cases, phages indirectly weaken the body’s defense mechanisms, allowing bacterial infection to spread.

Cases of direct negative effects of phages on immune cells are known. Sweere J.M. and colleagues in their studies showed that the lysogenic phage Pf, which infects *Pseudomonas aeruginosa*, is able to penetrate into murine and human leukocytes, where RNA is synthesized on the phage DNA, which induces the synthesis of Toll-dependent type I interferon. This cascade of reactions leads to the inhibition of tumor necrosis factor and the reduction of the phagocytosis of bacterial infection (Sweere et al., 2019).

The positive effect of phages on the immune system is that they can directly lyse pathogenic forms of bacteria without causing an infection or the development of inflammatory reactions. Barr J. and colleagues in their studies showed that endogenous bacteriophages actively inhabit mucous membranes where Ig-like domains of phages bind to mucin glycoproteins and form an active protective barrier against bacterial infections (Barr et al., 2013).

The structure of bacteriophages consists of a complex of proteins of different structures and nucleic acids, which endows them with antigenic properties. In addition, many bacteriophages have proteins similar to proteins of eukaryotic viruses. For example, a capsid protein HK97 that is also present in *Herviviricetes* viruses was found in the structure of *Caudoviricetes* phages (Dai and Zhou, 2018); many viruses of the *Poxviridae* and *Adenoviridae* families have a conserved capsid protein in their structure, which can be found in the structure of phages of the *Tectiliviricetes* class (Yutin et al., 2018). Therefore, when phage particles enter the blood or lymph, they can directly affect immune cells and trigger mechanisms of innate and adaptive immunity.

The first line of defense against pathogen invasion is innate immunity cells, neutrophils, and granulocytes, which recognize and destroy foreign agents and, if necessary, synthesize a spectrum of cytokines that enhance the development of adaptive immunity. In the 1960s, it was experimentally proved that leukocytes are able to absorb phage particles by endocytosis and lysate them (Kurzepa et al., 2009). In addition, it was found that bacteriophage nucleic acids released during the destruction of phage particles are pathogen-associated molecular structures (PAMP) for the immune system. PAMPs, in turn, trigger a cascade of biochemical reactions leading to the activation of cells of innate immunity (Roach et al., 2017).

Adaptive immunity is mainly developed to capsid proteins of bacteriophages. These proteins differ in their structure and immunogenicity and, as a consequence, induce different immune reactions of varying degrees of intensity. A sufficiently rapid formation of a large number of phage-specific antibodies can lead to neutralization of phage particles, which significantly reduces the effectiveness of antibacterial therapy, especially in the case of repeated administration of bacteriophages (Dąbrowska et al., 2014).

It was shown that different types of bacteriophages have different effects on the development of adaptive immunity factors. For example, the administration of purified *Staphylococcus* phages has a co-stimulatory effect on the CD3 protein complex and thereby increases the activity of human T cells. In contrast, purified T4 phage after administration inhibits the proliferation of human T cells activated by the CD3 complex (Górski et al., 2012).

Thus, in addition to the direct influence of bacteriophages on the qualitative and quantitative composition of bacterial populations due to lysing activity, they also have the ability to influence the development of factors of innate and adaptive immunity. Therefore, when using phage therapy in clinical practice, a clearer and more complete understanding of what effect a particular bacteriophage strain will have on the patient's immune system is necessary.

The aim of the presented work was to study the activity of gene expression (TLR3, TLR9, IFNγ, IL-2, IL-4, IL-5, and IL-6) and synthesis of cytokines (IFNγ, IL-2, IL-4, IL-5 and IL-6) responsible for the development of immune response, as well as investigate the levels of phage-specific IgA, IgG, IgM.

## 2 Materials and methods

### 2.1 Bacteriophage and bacterial strains used

In this work we used three previously isolated lytic bacteriophages of *E. coli* from our collection: vB_EcoM_SCS4 (*Caudoviricetes, Straboviridae, Tevenvirinae, Tequatrovirus*), vB_EcoS_SCS44 (*Caudoviricetes, Guernseyvirinae, Kagunavirus*) and vB_EcoM_SCS57 (*Caudoviricetes, Straboviridae, Tevenvirinae, Mosigvirus*). All the bacteriophages were isolated from wastewater collected from the territory of the Republic of Kazakhstan. The initial titer of phage lysates used was 10^8^ viral particles per 1 ml (Alexyuk et al., 2022).

### 2.2 Bacteriophage propagation

For the propagation of bacteriophages vB_EcoM_SCS4, vB_EcoS_SCS44 and vB_EcoM_SCS57, 80 ml of sterile Nutrient Broth (Conda, Spain) was poured into sterile 100 ml tubes, 2 ml of the host bacteria culture was added in the exponential growth phase and incubated at 37 °C until a turbidity index of 1 McF (3 × 10^8^ CFU/ml) was obtained. 8 ml of phage lysate of the propagated bacteriophage was added to the obtained bacterial suspension and cultivated at 37 °C for 24 h. After cultivation, the phage lysates were purified by centrifugation for 30 min at 4000 × *g* and filtration through 0.45 μm membrane filters (Montso et al., 2019). Bacteriophage titer in the obtained phage lysates was determined by the double-layer agar method (Gratia method) (Gratia, 1936). As a result, 90 ml of microbial cell-free phage lysate of each bacteriophage was obtained. The bacteriophage titers of vB_EcoM_SCS4, vB_EcoS_SCS44 and vB_EcoM_SCS57 in the phage lysates were 5.2 ± 0.6 × 10^8^, 3.4 ± 0.4 × 10^8^ and 3.8 ± 0.2 × 10^8^ PFU/ml, respectively.

### 2.3 Concentration of phage lysates

Phage lysates of bacteriophages vB_EcoM_SCS4, vB_EcoS_SCS44 and vB_EcoM_SCS57 were concentrated by ultrafiltration on Vivaspin (Sartorius, Germany) centrifugal concentrators (PES membrane, 30 kDa MWCO) to a final volume of 9 ml. As a result of the ultrafiltration, each phage lysate was concentrated 10-fold (Hietala et al., 2019). The bacteriophage titers of vB_EcoM_SCS4, vB_EcoS_SCS44 and vB_EcoM_SCS57 in the concentrated samples were 4.1 ± 0.3 × 10^9^, 6.2 ± 0.2 × 10^9^ and 3.3 ± 0.5 × 10^9^ PFU/ml, respectively.

### 2.4 Purification of phage lysates from extracellular bacterial nucleic acids

Then, 1 ml of 10x DNase I buffer, 20 µl of DNase I (1 unit/µl) and 20 µl of RNase A (10 mg/ml) were added to the obtained 9 ml of phage lysate; the resulting mixture was incubated at 37 °C for 90 min. After incubation, enzymatic activity was blocked by adding 400 μl of 0.5 M EDTA (the final concentration made 20 mM) (Jakočiūnė and Moodley, 2018).

### 2.5 Cesium chloride gradient ultracentrifugation

The phage-containing samples were purified from endotoxins by ultracentrifugation in a CsCl gradient. CsCl was mixed with sterile Tris–HCl buffer to obtain three concentrations: 1.3 g/mL, 1.5 g/mL, and 1.6 g/mL. The resulting CsCl concentrations were evenly layered into 14 mL thin-walled clear tubes designed for ultracentrifugation (Certified Free & Sterile Ultra-Clear Open-Top, Beckman Coulter), followed by the addition of approximately 4 mL of the phage-containing samples to each. The centrifugation was performed at 100,000 × *g* for 2 h in a SW40 Ti bacteriophage rotor (Beckman) on an Optima XPN 80 ultracentrifuge (Beckman). The purified bacteriophages were extracted by puncturing the tube with a syringe and picking out the whitish-gray band. The extracted material was dialyzed against the 1000-fold volume of SM buffer at 4 °C for 3 h, with the replacement of the full volume of the buffer after 1.5 h of dialysis. After the dialysis, the volume of phage-containing samples was brought to 4 mL with sterile SM buffer and passed through a bacterial filter with a pore diameter of 0.45 μm. The final phage titer was determined by the Gratia method (Carroll-Portillo et al., 2021).

### 2.6 Endotoxin level determination

Endotoxin levels in samples were determined using a commercial EndoLISA® kit (Biomérieux). Reagents were prepared and the entire assay was performed according to the manufacturer's instructions. Fluorescence was measured on an Infinite M200 Pro multi-plate spectrophotometer (TECAN): excitation wavelength - 380 nm, luminescence wavelength - 445 nm. Measurements were made immediately after applying the solution and after 90 min of incubation at 37 °C. All the tests were performed in two replicates; the results were calculated using the calibration curve (Szermer-Olearnik and Boratyński, 2015). Endotoxin levels in vB_EcoM_SCS4, vB_EcoS_SCS44 and vB_EcoM_SCS57 bacteriophage samples after the affinity chromatography averaged 0.1 ± 0.003 EU/mL.

### 2.7 Experiments on animals

Male BALB/c mice aged 4–5 weeks, free of specific pathogens, were used in the studies. The mice were acclimatized to vivarium conditions for 2 weeks before the experiment, kept in individually ventilated cages with 7 individuals in each cage, in a 12-hour light-dark cycle at a temperature of 20–25°С. The mice had unrestricted access to standard rodent food and water. Each experimental group included 14 animals. Bacteriophage and control samples (sterile PBS, pH 7.4) were injected into the abdominal cavity of mice at 200 μl per mouse. The concentration of bacteriophages in the samples averaged 10^8^ PFU /mL.

Three days after the first bacteriophage injection, four mice from each group were euthanized (euthanasia was performed according to AVMA recommendations using a CO_2_ inhalation agent), and peritoneal leukocytes were collected by washing the abdominal cavity with Dulbecco's phosphate-salt buffer without calcium and magnesium. The resulting cell suspensions were centrifuged at 1000 × *g*, and the supernatant was removed (Ray and Dittel, 2010). The resulting precipitate was resuspended in PBS at a concentration of 2 × 10^6^ cells/mL, aliquoted and frozen.

After 3 weeks, the remaining mice were given a bacteriophage booster injection; a week later, blood was collected from all the mice. The blood samples were incubated at 37 °C for 30 min and centrifuged at 4000 × *g* for 30 min to precipitate the formed elements. The resulting sera were carefully withdrawn without stirring the precipitated cells, aliquoted 30 μL each and frozen.

### 2.8 Isolation of RNA from peritoneal leukocytes

Total RNA was isolated from peritoneal leukocytes using a commercial Rneasy Mini Kit for RNA extraction (QIAGEN, Germany); all the procedures were performed according to the manufacturer's instructions.

### 2.9 Reverse transcription reaction

Reverse transcription was performed using TaqMan® Reverse Transcription Reagents (ThermoFisher Scientific, USA) in 20 µl of the reaction mixture containing DEPC-treated water – 1.6 µl, 10X RT Buffer – 2 µl, 25 mM MgCl2 – 1.4 µl, 10 mM dNTP mix (2.5 mM each) – 4 µl, RNase Inhibitor (20 U/µL) – 1 µl, MultiScribe™ RT (50 U/µL) – 1 µl, 50 µM Oligo d(T)16 – 1 µl and Template RNA - 8 µl. The reaction was carried out as follows: a mixture of Oligo d(T)16 primer with RNA was heated at 65 °C for 5 min, cooled at 4 °C for 2 min, and then the remaining mixture of reagents was added to the mixture of RNA and Oligo d(T)16 primer. Conditions: 30 min at 37 °C, 5 min at 95 °C.

### 2.10 Determination of gene expression levels

Taking into account the fact that bacteriophages are viruses, the criterion for selecting the studied immune response factors was their influence on the development of antiviral immunity. TLR3, TLR9 were selected as key links of innate antiviral immunity capable of recognizing virus nucleic acids. Activation of these receptors triggers a cascade of biochemical reactions leading to the development of antiviral immune response. IFNγ is a key cytokine in the development of adaptive immunity, it activates signaling pathways leading to the development of antiviral immune response. IFNγ possesses the ability to directly inhibit viral replication in infected cells. IL-2 is the main cytokine regulating the growth, proliferation and differentiation of T-lymphocytes, it stimulates the development of NK-cells, which leads to the development of cellular antiviral immunity. IL-6 plays a key role in the development of immunity and inflammatory responses, it promotes the proliferation and differentiation of T- and B-lymphocytes, resulting in the development of an overall adaptive immune response. IL-5 is the main cytokine that activates eosinophils and leads to allergic reactions. Data on the level of expression and synthesis of this interleukin will allow us to assess the allergenicity of the bacteriophages under study, which is important in the conditions of their use as antibacterial therapeutic agents (Akira et al., 2006; Dinarello, 2007).

The levels of expression of marker genes TLR3, TLR9, IFNγ IL-2, IL-4, IL-5, and IL-6 was determined by TaqMan™ Gene Expression Assay in real-time PCR (Applied Biosystems) in accordance with the manufacturer's protocol. A TaqMan real-time PCR assay was performed in 20 µl of the reaction mixture (Fast Advanced Mix for PCR - 10 µl, TaqMan probe - 1 µl, deionized water - 6 µl). Aliquots of cDNA (3 μl each) were added to the wells of the plate containing the reaction mixture and sealed with an optical film, followed by centrifugation to precipitate drops. A plate (strip) was installed in a cycler (PikoReal 96, ThermoScientific), and the location, characteristics of the samples and dye used were noted in the program. The PCR assay was performed: 95 °C - 20 s.; 95°С - 1 min.; 60°С - 20 s.; 40 cycles. Normalization of gene expression was carried out using the housekeeping genes actin and Rn18s. The results were calculated using the 2^−∆∆CT^ method (Livak and Schmittgen, 2001; Bustin et al., 2009).

### 2.11 Determination of cytokine concentration

The concentration of cytokines in mouse sera was determined by the ELISA method using the following commercial kits: Mouse IL-2 ELISA Kit, Mouse IL-4 ELISA Kit, Mouse IL-5 ELISA Kit, Mouse IL-6 ELISA Kit and Mouse IFN-γ ELISA Kit (Invitrogen). Standards, buffer solutions, and the whole course of the assay were according to the manufacturer's instructions. The concentration of the studied cytokines was determined in the serum of each mouse; all the tests were performed in two replicates. The results were calculated according to the calibration curve.

The titer of phage-specific IgA, IgG, IgM in mouse sera was determined with ELISA. First, 100 µl of each of the purified bacteriophages vB_EcoM_SCS4, vB_EcoS_SCS44 and vB_EcoM_SCS57 diluted 200 times in sample dilution buffer from the kits was applied to the wells of a sterile microplate and incubated at 4 °C for 18 h to obtain plates with specific antigens to phage-specific antibodies. The rest of the test procedures were carried out according to the standard methodology (Natarajan and Remick, 2013). Commercial preparations of species-specific antibodies labeled with horseradish peroxidase (Southern Biotechnology Associates, Inc., USA) were used in the experiment. The antibody titer was defined as the highest antibody dilution at which the absorbance level was above the cutoff line.

### 2.12 Statistics and graphic display of results

Statistical processing of the data was performed using Microsoft Excel, Microsoft Office package was used for tabular and graphical presentation of the results. The values of all parameters were expressed as mean with standard deviation (SD). Significant differences between the results of experimental and control groups were determined using 2-tailed unpaired Student's *t-*test and one-factor analysis of variance with Tukey HSD (Tukey HSD) post hoc test. P -values <0.05 were considered statistically significant.

## 3 Results

### 3.1 Gene expression

**Fig. 1:**
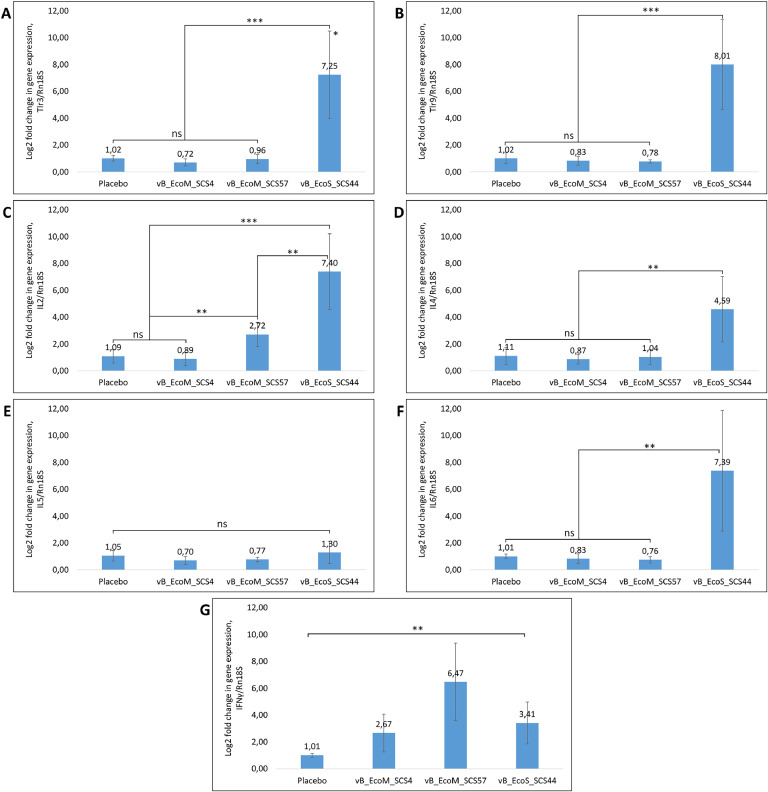
Activity of Tlr3 (A), TLR9 (B), IL-2 (C), IL-4 (D), IL-5 (E), IL-6 (F) and IFNγ (G) gene expression after the intraperitoneal injection of *E. coli* lytic bacteriophages vB_EcoM_SCS4, vB_EcoS_SCS44, vB_EcoM_SCS57 in mice. * Standard deviation of 2-∆∆Ct values, ** *P* ≤ 0.05, *** *P* ≤ 0.01, ns - not significant.

After determining the relative changes in the expression of the studied genes, we found that introduction of the vB_EcoM_SCS4 bacteriophage did not cause any significant changes in the expression of TLR3, TLR9, IL-2, IL-4, IL-5 and IL-6 genes that made 0.72 ± 0.2, 0.83 ± 0.3, 0.89 ± 0.5, 0.87 ± 0.3, 0.7 ± 0.3 and 0.83 ± 0.3 log2 fold, respectively (Fig. 1A–F), but increased the expression of IFNγ by 2.67 ± 1.4 Log2 fold (Fig. 1G). The administration of the vB_EcoS_SCS44 bacteriophage to mice resulted in a significant increase in the expression of TLR3, TLR9, IL-2, IL-4, IL-6 and IFNγ genes by 7.25 ± 3.2, 8.01 ± 3.3, 7.4 ± 2.8, 4.59 ± 2.4, 7.39 ± 4.4 and 3.41 ± 1.5 Log2 fold, respectively, (Fig. 1A–G) and had a minor effect on the IL-5 gene expression activity amounting to 1.3 ± 0.8 Log2 fold (Fig. 1E). Introduction of the vB_EcoM_SCS57 bacteriophage increased the expression of IL-2 and IFNγ genes by 2.72 ± 0.9 and 6.47 ± 2.8 Log2 fold, respectively (Fig. 1D and G), and had no significant effect on the activity of TLR3, TLR9, IL-4, IL-5 and IL-6 genes expression which was 0.96 ± 0.3, 0.78 ± 0.1, 1.04 ± 0.5, 0.77 ± 0.1 and 0.76 ± 0.2 Log2 fold compared to the control group (Fig. 1A,B,C,E and F).

Thus, bacteriophage vB_EcoS_SCS44 had the maximum stimulatory effect, increasing the expression of almost all cytokines studied, except IL-5, by 3–8 times. Injection of bacteriophages vB_EcoM_SCS4 and vB_EcoM_SCS57 into mice resulted in an insignificant stimulating effect, increasing the expression of IFNγ genes by 3 and 6 times, respectively, and in the case of phage vB_EcoM_SCS57 by 3 times the expression of IL-2 genes.

### 3.2 Cytokine levels

**Fig. 2:**
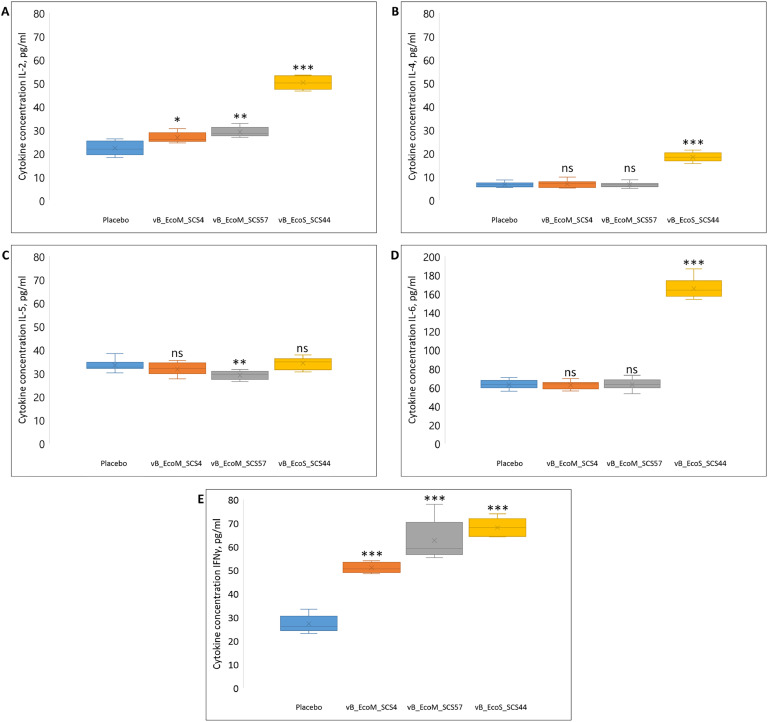
Concentration of IL-2 (A), IL-4 (B), IL-5(C), IL-6(D) and IFNγ (E) in mouse sera after the intraperitoneal injection of *E. coli* lytic bacteriophages vB_EcoM_SCS4, vB_EcoS_SCS44 and vB_EcoM_SCS57 in mice. P - values are shown when compared with the placebo group: * *P* ≤ 0.05, ** *P* ≤ 0.01, *** *P* ≤ 0.001.

ELISA results showed that two intraperitoneal administrations of purified bacteriophages vB_EcoM_SCS4 and vB_EcoM_SCS57 did not significantly change the levels of IL-2 (26.8 ± 2.3 and 29.1 ± 2.2 pg/mL), IL-4 (6.9 ± 1.6 and 6.5 ± 1.1 pg/mL), IL-5 (31.8 ± 2.6 and 29.2 ± 1.8 pg/mL) and IL-6 (62.3 ± 4.3 and 63 ± 6.1 pg/mL) compared with the controls (IL-2 – 22.3 ± 3.1, IL-4 – 6.6 ± 1.1, IL-5 – 33.4 ± 2.4, IL-6 – 62.8 ± 4.5 pg/mL) (Fig. 2A–D). However, the levels of IFNγ in the sera of mice increased approximately 2 and 2.5-fold (51.1 ± 2.3 and 62.6 ± 8.9 pg/mL) after the administration of these bacteriophages compared to the control group (27.2 ± 3.8 pg/mL) (Fig. 2E).

The administration of the purified bacteriophage vB_EcoS_SCS44 resulted in a 2-fold increase in the serum concentration of IL-2 (50.2 ± 2.9 pg/mL) in mice (Fig. 2A) and approximately a 2.5-fold increase in the concentration of IL-4 (18.3 ± 1.8 pg/mL), IL-6 (165.3 ± 10 pg/mL) and IFNγ (68.1 ± 4 pg/mL) (Fig. 2B, D and E) compared with the controls. IL-5 concentration remained at about the same level (34.3 ± 2.5 pg/mL) as in the control animals (Fig. 2C).

**Fig. 3:**
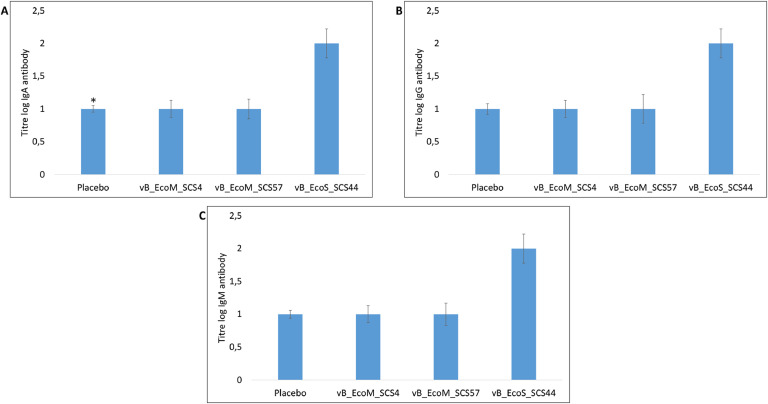
Concentration of IgA (A), IgG (B) and IgM(C) in mouse sera after the intraperitoneal injection of *E. coli* lytic bacteriophages vB_EcoM_SCS4, vB_EcoS_SCS44 and vB_EcoM_SCS57 in mice.* - standard deviation.

Thus, the obtained data on the concentration of IL-2, IL-4, IL-5, IL-6 and IFNγ correspond to the results of expression of genes encoding these cytokines. The phage vB_EcoS_SCS44 had the maximum stimulatory effect, the administration of which resulted in a 2 - 3-fold increase in the concentration of IL-2, IL-4, IL-6 and IFNγ in the serum of mice. Intraperitoneal injection of vB_EcoM_SCS4 and vB_EcoM_SCS57 phages into mice resulted in an approximately 2-fold increase in the concentration of IFNγ only. No significant differences in the concentration of IL-5 in the sera of mice of the experimental groups from the control group were recorded (Fig. 3).

### 3.3 Phage-specific Ig levels

ELISA results showed that after two intraperitoneal administrations of purified bacteriophages vB_EcoM_SCS4 and vB_EcoM_SCS57 to mice, the titer of phage-specific IgA, IgG and IgM was comparable to the titer of immunoglobulins in the sera of the control animals. The administration of bacteriophage vB_EcoS_CS44 led to the formation of the titer of specific IgE, IgG and IgM that was 2 times higher than the titer of these immunoglobulins in the control animals.

Thus, it was shown that bacteriophage vB_EcoS_CS44 possessed immunogenic activity and stimulated the synthesis of phage-specific antibodies in mice' serum after intraperitoneal administration, while phages vB_EcoM_SCS4 and vB_EcoM_SCS57 were neutral, and their administration did not stimulate the synthesis of phage-specific antibodies in mice.

## 4 Discussion

It was commonly believed that bacteriophages have no significant direct effect on eukaryotic cells because they are unable to interact with their cell receptors. However, at present, there are works describing interactions between bacteriophages and eukaryotic cells (Podlacha et al., 2021; Porayath et al., 2018; Hodyra-Stefaniak et al., 2015). Therefore, additional studies on the direct effect of phages on human and animal immune response reactions are necessary for widespread use and a more effective application of phage therapy methods. The presented work shows the results of studies of immune response reactions to intraperitoneal injection of lytic *E. coli* bacteriophages in mice.

In case of bacteriophage accumulation, the obtained phage lysates contain many fragments of destroyed bacterial cells, including parts of cell walls, endotoxins and nucleic acids, which are strong immunostimulants (Blander and Barbet, 2018; Kimbrell et al., 2008). Therefore, to confirm that bacteriophages directly lead to the development of immune reactions, phage-containing samples pretreated with nucleases and purified by ultracentrifugation in a CsCl gradient were used in the experiments.

Among the available routes of administration, oral delivery is considered the simplest and least invasive for bacteriophages used as therapeutic agents. However, it must be taken into account that samples introduced in this way are subject to the effect of the first pass through the liver, in addition, bacteriophages can be inactivated by the acidic environment of the stomach. In our studies, we chose the method of introducing bacteriophages by intraperitoneal injection, which is not only less traumatic for mice, in contrast to intravenous administration, but also allows for a reduction in dose variability (Turner et al., 2011). In addition, when administered through the abdominal cavity, bacteriophages penetrate into the vascular bed, providing a systemic effect comparable to intravenous administration and is optimal for assessing immune response reactions, especially reactions of peritoneal leukocytes (Dabrowska, 2019; Dubos et al., 1943).

Considering all the advantages of intraperitoneal administration, it should be noted that this method is extremely rare in clinical practice, unlike oral and intravenous, it does not allow modeling the mucosal immune response that forms with oral administration, therefore the obtained immunological data may have limited translational value (Al Shoyaib et al., 2019). To overcome this limitation, in our further studies, we plan to expand the range of methods for administering bacteriophages to determine their immunomodulatory properties.

Three days after the intraperitoneal injection of the studied bacteriophages into mice, the expression levels of the genes responsible for the factors of innate (TLR3, TLR9), adaptive general (IL-6), cellular (IL-2, IFNγ), humoral immunity and allergic reaction development factors (IL-5) were determined in peritoneal leukocytes (Akira et al., 2006; Dinarello, 2007).

It was found that phage vB_EcoS_SCS44 had the highest immunogenicity, and its administration increased the expression of TLR3, TLR9, IL-2, IL-4, IL-6 and IFNγ genes many-fold. The administration of bacteriophage vB_EcoM_SCS57 resulted in a 3-fold increase in the IL-2 gene expression and a 6.5-fold increase in the IFNγ gene expression. In the case of phage vB_EcoM_SCS4, a two-fold increase in the expression of IFNγ gene only was observed. The administration of the studied bacteriophages did not significantly affect the expression of IL-5 gene.

Although TLR3 is mainly responsible for detecting RNA viruses, there is evidence that TLR3 can indirectly respond to DNA viruses as well. Thus, in their studies Sweere J.M. and colleagues showed that penetration of DNA phage Pf into leukocytes causes TLR3 activation and IFNα synthesis (Sweere et al., 2019). The authors explain this phenomenon by the synthesis of RNA on phage DNA, to which Tol-dependent receptors respond, or by the presence of RNA molecules in the phage capsid that accidentally got there during assembly. A similar finding was observed in our studies when the intraperitoneal injection of vB_EcoS_SCS44 DNA phage into mice resulted in an increased expression of TLR3 genes. However, it should also be noted that an increased expression of TLR9, IL-2, IL-4, IL-6 and IFNγ genes in peritoneal leukocytes was recorded upon administration of this phage. It can be concluded that the structure of phage vB_EcoS_SCS44 contains elements that make it an immunogenic agent that activates factors of innate, adaptive cellular, and humoral immunity.

Taking into account that IL-5 stimulates the proliferation and maintains the population of eosinophils – the primary cells involved in the development of allergic reactions and antiparasitic immunity – we can conclude that the studied bacteriophages are not allergens and, due to their nature, are not recognized by the immune system as parasites. At the same time, it is necessary to note an increase in the expression of IFNγ gene in all the mice injected with the phages. Apparently, this is due to the fact that bacteriophages are viruses and, by various mechanisms, are able to penetrate into eukaryotic cells (Nguyen et al., 2017), which leads to the induction of IFNγ, which plays a key role in the activation of apoptosis and the fight against intracellular pathogens such as viral infections.

The cytokine concentrations in the sera of mice showed that the highest level of IL-2, IL-4, IL-6 and IFNγ was recorded after the administration of phage vB_EcoS_SCS44 and were approximately 2 to 3 times higher compared with the control. The administration of phages vB_EcoM_SCS4 and vB_EcoM_SCS57 induced an increase in the level of IFNγ only. The IL-5 level in all the experimental groups remained approximately at the same level as in the control group.

Overall, the level of the studied interleukins reflects the level of expression of the corresponding genes and confirms the fact that the phage vB_EcoS_SCS44 had an immunomodulatory activity and was able to induce factors of both innate and adaptive immunity. Whereas, phages vB_EcoM_SCS4 and vB_EcoM_SCS57 had a minimal immunomodulatory activity and their intraperitoneal administration to mice stimulated only the IFNγ formation from the cytokines tested.

The effect of bacteriophages vB_EcoM_SCS4, vB_EcoS_SCS44 and vB_EcoM_SCS57 on the activity of phage-specific humoral immune response formation was evaluated based on the difference between the titer of phage-specific IgA, IgG and IgM in the sera of the mice after the administration of the purified phage-containing samples and the titer of the same immunoglobulins in the serum of the control mice.

As a result of these studies, it was found that the intraperitoneal injection of the purified bacteriophages vB_EcoM_SCS4 and vB_EcoM_SCS57 into mice resulted in the same titer of phage-specific immunoglobulins as in the controls. The administration of phage vB_EcoS_CS44 resulted in a two-fold increase in the titer of all the immunoglobulins tested. These results also confirm the presence of immunogenic activity in phage vB_EcoS_CS44 (Van Snick and Masson, 1980). On the basis of this, it can be assumed that vB_EcoS_CS44 phage will be neutralized rather quickly by the immune system of the patient, and its use as a therapeutic agent will be ineffective. In turn, phages vB_EcoM_SCS4 and vB_EcoM_SCS57, due to their low immunogenicity, will persist in the patient's body for a longer period of time without being neutralized by specific antibodies, which increases their efficacy against pathogenic *E.coli*.

The differences in immunomodulatory properties between the phages studied can apparently be explained based on their structural features. Thus, phage vB_EcoS_SCS44, which had the highest immunomodulatory activity, differed in structure from phages vB_EcoM_SCS4 and vB_EcoM_SCS57, being a sipho-like virus, whereas the other two phages were myo-like viruses. This assumption is consistent with a large number of studies that show that phages different in capsid and nucleic acid structure are able to exert different effects on the immune system (Champagne-Jorgensen et al., 2023; Miernikiewicz et al., 2013; Freyberger et al., 2018). For example, in their studies Sweere J.M. and colleagues showed that the administration of Pf4, a filamentous DNA phage that infects *P. aeruginosa*, to mice inhibited TNF synthesis and reduced the activity of all the inflammatory reactions in the body (Sweere et al., 2019). However, the work of Gogokhia L. et al. presented the results showing that the administration of a cocktail of different DNA phages to mice stimulated the synthesis of cytokines, the development of Th1-lymphocytes and an increase in the concentration of IFNγ (Gogokhia et al., 2019).

Studies have demonstrated that modifying bacteriophages can reduce their immunogenicity and prolong circulation time within the macroorganism. One of the first and classical approaches was the covalent modification of capsid proteins with polyethylene glycol, which allowed for an increase in circulation time and a significant decrease in the neutralization of phages by antibodies (Merril et al., 2003; Kim et al., 2008). The next step was site-directed mutagenesis, which allows changing the antigenic epitopes of capsid proteins, thereby reducing their recognition by the immune system (Ando et al., 2015; Pires et al., 2016; Cui et al., 2024). Phage masking and delivery methods also allow immune barriers to be overcome. For example, Kim et al. coated the bacteriophage capsid with a biodegradable PLGA polymer and a lipid layer, which masked immunogenic epitopes and prevented the development of phage-specific immune responses (Kim et al., 2022). Encapsulation of phages in liposomes, polymer nanoparticles and biopolymer-based microcapsules protects them from the aggressive environment of the gastrointestinal tract, immune cells and enzymes, preserving their lytic activity and increasing circulation time (Ma et al., 2012; Malik et al., 2017; Makhlouf et al., 2023)

To increase the circulation period of bacteriophages in the body, overcome immune barriers, expand the spectrum of sensitive bacterial strains and increase the overall effectiveness of phage therapy, the use of hybrid approaches seems most appropriate. Such strategies include the combined use of bacteriophages with traditional antibacterial preparations and immunostimulating adjuvants, genetically engineered modification of phages to alter their antigenicity or host specificity, the creation of multiphage cocktails, and the development of methods for protecting and targeted delivery of phages (encapsulation in liposomes, polymer or biopolymer systems) to bacterial targets (Cui et al., 2023; Shein et al., 2023). The combined use of these approaches allows us to overcome the key limitations of classical phage therapy, increase the stability and bioavailability of phages, and minimize the risk of developing resistance in pathogens.

## 5 Conclusion

Based on the results of the conducted work, it was established that of the three studied lytic bacteriophages of *E. coli*, vB_EcoM_SCS4 and vB_EcoM_SCS57 phages were not able to significantly affect the expression and level of the studied interleukins and induce the formation of antigen-specific immunoglobulins, while phage vB_EcoS_SCS44 had a pronounced immunostimulant activity. The differences in immunomodulatory properties of the studied phages likely depended on their peculiarities of structure since phage vB_EcoS_SCS44 has the morphotype of a siphovirus, and phages vB_EcoM_SCS4 and vB_EcoM_SCS57 are myoviruses. However, to confirm this hypothesis, further studies on immunomodulatory activity with a large sample of bacteriophages with different structures are needed.

## Funding

This work was supported by Ministry of Science and Higher Education of the Republic of Kazakhstan, grant number AP19679466.

## Ethics statements

All animal experiments were performed in accordance with the ARRIVE guidelines and the National Institutes of Health guidelines for the care and use of laboratory animals (NIH Publication No 8023, revised 1978). The ethical protocol was approved by the decision of the local ethical commission of the Research and Production Center for Microbiology and Virology LLP No 02–12–59 of 06/03/2024

## CRediT authorship contribution statement

**Madina S. Alexyuk:** Writing – review & editing, Writing – original draft, Visualization, Validation, Project administration, Methodology, Investigation, Funding acquisition, Formal analysis, Conceptualization. **Andrey P. Bogoyavlenskiy:** Writing – review & editing, Methodology, Formal analysis, Conceptualization. **Irina A. Zaitseva:** Investigation, Formal analysis, Data curation. **Elmira S. Omirtaeva:** Investigation, Formal analysis, Data curation. **Yergali S. Moldakhanov:** Investigation, Formal analysis, Data curation. **Kuralay S. Akanova:** Investigation, Formal analysis, Data curation. **Elmira I. Anarkulova:** Investigation, Formal analysis, Data curation. **Vladimir E. Berezin:** Writing – review & editing, Supervision. **Pavel G. Alexyuk:** Writing – review & editing, Writing – original draft, Visualization, Validation, Methodology, Investigation, Formal analysis, Conceptualization.

## Declaration of competing interest

The authors declare that they have no known competing financial interests or personal relationships that could have appeared to influence the work reported in this paper.

## Data Availability

Data will be made available on request.
